# Isolated gallbladder metastasis of melanoma: Case report

**DOI:** 10.1016/j.ijscr.2020.04.086

**Published:** 2020-05-14

**Authors:** Gonzalo Guido D'Urso Vilar, Facundo Iriarte, Daniela Speisky, Mariano Luis Bregante, Sergio Damian Quildrian

**Affiliations:** a General Surgery Department, British Hospital of Buenos Aires, Perdriel 74, CABA, 1280, Argentina; b Pathology Department, British Hospital of Buenos Aires, Perdriel 74, CABA, 1280, Argentina

**Keywords:** Melanoma, Isolated metastasis, Gallbladder, Laparoscopic cholecystectomy, Case report

## Abstract

**Background:**

Although metastatic melanoma is most frequently found in liver, lungs, and brain, most metastases found in the gallbladder are from melanoma. Here, we present a case of isolated metastatic melanoma found during cholecystectomy.

**Presentation of case:**

74-year-old male with a personal history of hypertension, diabetes mellitus, obesity, and arrhythmia. A skin lesion was found on the right malar region. An excisional biopsy was performed and histopathological examination showed an ulcerated nodular-type malignant melanoma, Breslow 7.6 mm, Clark IV. Surgical excision with margins of 2 cm and sentinel lymph-node biopsy was carried and were negative. Abdominal sonography at 6 months showed an 18 mm solid mass adhered to the wall of the gallbladder that was suggestive of a polyp. Thorax-abdomen-pelvis CT showed no abnormalities. The gallbladder lesion had increased in volume on the following sonography and therefore, cholecystectomy was performed. Histopathological study revealed melanoma infiltrating the mucosa and muscular layer. Written informed consent was previously obtained, and Institutional Review Board approval was not needed.

**Discussion:**

Isolated metastatic melanoma in the gallbladder is uncommon. Follow-up controls with images are important in the diagnosis. As most metastatic melanoma to the gallbladder are asymptomatic, surgeons should have high level of suspicion. Cholecystectomy could prolong survival in these patients.

**Conclusion:**

Isolated gallbladder metastasis of melanoma is an uncommon presentation of this disease. Laparoscopic cholecystectomy is an adequate procedure in this particular situation and may improve patient survival. The presentation of this case may help surgeons to maintain a high level of suspicion regarding the condition.

## Highlights

• Isolated gallbladder metastasis of cutaneous malignant melanoma.
• Laparoscopic cholecystectomy of gallbladder with isolated metastasis.
• Unusual case successfully treated with laparoscopic resection after imaging control.

## 1 Introduction

Metastatic melanoma is frequently found in liver, lung, and brain [1]. In the gastrointestinal tract, metastases are most commonly found in the small bowel (35–65%), colon (5–9%), and stomach (5–7%) [2]. Primary malignant melanoma of the gallbladder is extremely rare with only case reports published in the literature. Moreover, isolated gallbladder metastasis from malignant melanoma is even more unusual [3]. Gallbladder metastasis is generally asymptomatic or may present as cholecystitis and is therefore typically found during follow-up on imaging studies [4] or after cholecystectomy [5]. Most of the times, its primary origin is unknown. Here we present a case of isolated gallbladder metastasis of malignant melanoma found after cholecystectomy. This case report has been reported in line with the SCARE checklist [6].

## 2 Presentation of case

**Fig. 1:**
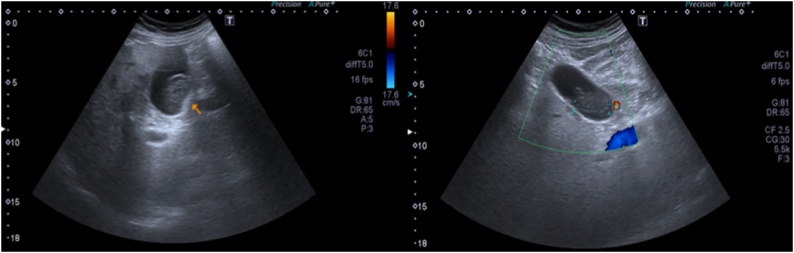
US: Solid image adhered to the wall of the gallbladder.

The patient was a 74-year-old male with a personal history of hypertension, type II diabetes mellitus, obesity, and arrhythmia, who presented with a skin lesion in the right malar region. An excisional biopsy was performed and histopathological study was consistent with an ulcerated nodular malignant melanoma, Breslow 7.6 mm, Clark IV. Mitosis was more than 1 per mm^2^, with no signs of vascular or perineural involvement and no signs of regression. Resection margins were negative. Thyroid and cervical sonography and thorax-abdomen-pelvis computed tomography scan (TAP CT) were negative. Complete resection with 2 cm surgical margins and sentinel lymph-node biopsy were performed without residual lesion or lymphatic metastasis. Three months later, TAP CT and thyroid and cervical sonography (US) were negative. At seven months of follow-up, thorax x-rays were requested and no distant metastasis was found. Abdominal US revealed a solid image adhered to the wall of the gallbladder suggestive of a polyp measuring 18 × 16 mm (Fig. 1). At 10 months of follow-up, a TAP CT was performed showing no evidence of disease. Thus, 3 months later (13 months postoperatively (POP)) abdominal US and thorax x-rays were performed of which the former showed a thin gallbladder wall with a heterogeneous solid mass inside measuring 34 × 20 × 24 mm. The bile ducts were spared. Magnetic resonance imaging (MRI) of the abdomen could not be performed because of a non-MRI-conditional pacemaker of the patient. Therefore, a CT scan was performed which revealed a thin-walled gallbladder with high-density content. There was no liver metastasis and no changes were observed compared with the previous CT scan. One month later (15th month POP), a laparoscopic cholecystectomy was carried out successfully and the patient was discharged the following day.

Macroscopic examination showed a solid brown polyp measuring 3.5 × 3 cm located in the fundus of the gallbladder.

The histological sections of material embedded in paraffin and colored with hematoxylin and eosin demonstrated numerous atypical cells with a dense eosinophilic and heavily pigmented cytoplasm and nuclei with granular chromatin. The lesion involved the gallbladder mucosa and muscle layers. Complete resection with tumor-free margins had been achieved.

Immunohistochemistry was performed on histological sections of 3 microns by means of an automated system according to the manufacturer’s guidelines (Benchmark XT, Ventana).

**Fig. 2:**
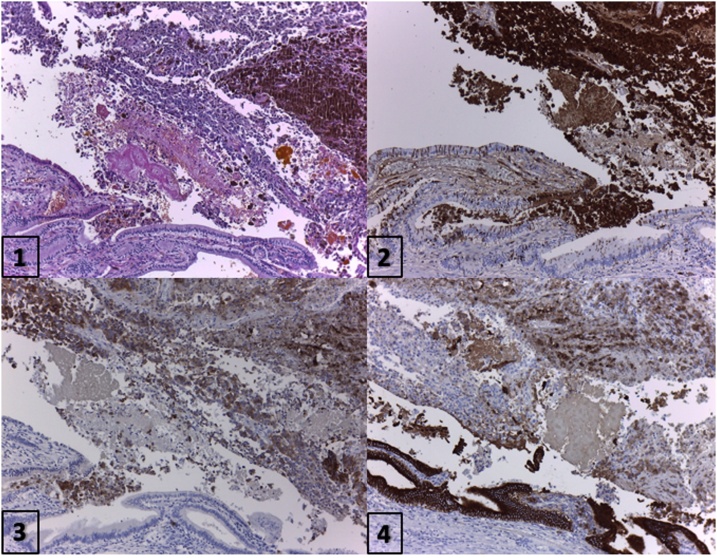
Pathology 1) HyE 2) S100 3) Melan A 4) Cytokeratin AE1AE3.

The neoplastic cell population showed intense positivity for S100 protein, Melan-A, and Sox10. The cytokeratin cocktail (Cytokeratin AE1AE3) was negative in the tumor component (Fig. 2).

The histopathological findings together with the immune profile were consistent with melanoma.

**Fig. 3:**
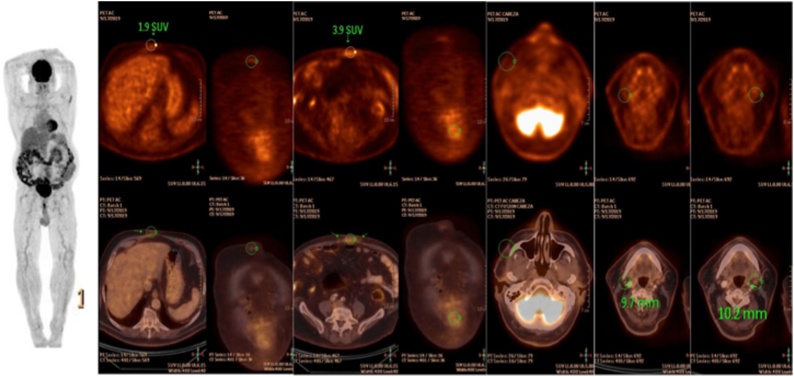
PET-TC showed no abnormalities.

The patient recovered without complications and the following month (16th month POP) a PET CT was performed with negative results (Fig. 3). Two months post-cholecystectomy, the patient was started on nivolumab therapy, and currently, the patient has completed a follow-up of 7 months after cholecystectomy and 22 months after cutaneous melanoma resection without evidence of disease.

## 3 Discussion

Cutaneous melanoma metastases are most frequently found in liver, lungs, and brain, although they may be found almost anywhere in the body [1]. Isolated metastasis from melanoma is uncommon with only case reports published in the literature. Although primary gallbladder melanoma seems to be even more unusual, it remains a debatable clinical entity. In 1998, Dong et al. presented 19 cases of melanoma of the gallbladder registered since 1970 out of more than 11,500 patients with the disease; however, only three of them were isolated metastasis [3]. Subsequently, only few cases were reported denoting the extremely low incidence of this condition [4,5]. In our case, previous images showed no other metastases, at least not in thorax, abdomen, and pelvis, and after surgery, a PET scan was also negative for metastasis.

Although most metastatic melanomas of the gallbladder are asymptomatic, different authors reported that 50% or more of their patients had symptoms that preceded the diagnosis [3,7]. Patients with gallbladder metastasis may present with epigastric or right upper quadrant pain, sometimes mimicking acute cholecystitis [2,3,5,8]. In symptomatic as well as asymptomatic patients, abdominal US is the imaging study of choice to assess metastasis in the gallbladder. Due to their low density, tumor masses in this location do not produce acoustic shadowing, sometimes mimicking polypoid lesions. Therefore, Doppler ultrasonography may be useful to characterize the lesion [9]. In our case, both CT scan and US were performed; however, US first detected the gallbladder lesion and also provided a better description of the entity. Although the value of imaging studies is controversial for the follow-up of cutaneous melanomas with localized disease, in cases of high-risk disease (Breslow over 4 mm), as was our case, imaging studies may be useful when assessing disease progression [10].

Even though a consensus has not been established because of the rarity of the condition, different studies have reported good outcomes and better survival in patients with isolated metastatic melanoma in the gallbladder after cholecystectomy [3,7]. Tuveri presented a case with a 5-year disease-free survival after laparoscopic cholecystectomy for isolated gallbladder metastasis [11], and Dong reported a case with a POP survival of as long as 13.8 years [3].

The “gold-standard” treatment of metastatic melanoma of the gallbladder remains unclear. A recent paper encourages an open rather than laparoscopic surgical approach in order to avoid trocar recurrence and to detect other gastrointestinal metastases that went undetected preoperatively [12]. Nevertheless, Tuveri reported a 5-year survival after laparoscopic cholecystectomy [9], and this finding is supported by other authors [13,14]. Our patient underwent laparoscopic cholecystectomy, was discharged home on the following day, and recovered uneventfully.

## 4 Conclusion

Isolated gallbladder metastasis of cutaneous melanoma is an uncommon presentation of this disease. Follow-up controls with imaging studies are important in the diagnosis of asymptomatic patients. We believe that laparoscopic cholecystectomy is an adequate procedure in this particular situation and may improve patient survival. The presentation of this case may help surgeons to maintain a high level of suspicion regarding the condition.

## Declaration of Competing Interest

Gonzalo G. D’Urso Vilar, Facundo Iriarte, Daniela Speisky, Mariano L. Bregante and Sergio D. Quildrian declare that there is no conflict of interest regarding the publication of this article.

## Funding

There were no sources of funding.

## Ethical approval

Ethical approval has been exempted by our institution.

## Consent

Written informed consent was obtained from the patient for publication of this case report and its accompanying images.

## Registration of research studies

None.

## Guarantor

Sergio D. Quildrian accepts full responsibility for the article.

## Provenance and peer review

Not commissioned, externally peer-reviewed.

## CRediT authorship contribution statement

**Gonzalo Guido D'Urso Vilar:** Conceptualization, Methodology, Investigation, Supervision, Writing - original draft. **Facundo Iriarte:** Conceptualization, Methodology, Supervision, Visualization, Writing - original draft, Writing - review & editing, Investigation. **Daniela Speisky:** Visualization, Resources, Writing - original draft, Writing - review & editing. **Mariano Luis Bregante:** Validation, Supervision, Project administration, Writing - review & editing. **Sergio Damian Quildrian:** Validation, Supervision, Project administration, Writing - review & editing.
